# Modelling a Spectral Index to Detect Dispersed Oil in a Seawater Column Depending on the Viewing Angle: Gulf of Gdańsk Case Study

**DOI:** 10.3390/s20185352

**Published:** 2020-09-18

**Authors:** Emilia Baszanowska, Zbigniew Otremba, Jacek Piskozub

**Affiliations:** 1 Department of Physics, Gdynia Maritime University, 81-225 Gdynia, Poland; z.otremba@wm.umg.edu.pl; 2 Institute of Oceanology, Polish Academy of Sciences, Powstańców Warszawy, 81-712 Sopot, Poland; piskozub@iopan.gda.pl

**Keywords:** oil-in-seawater emulsion, oil detection, radiance reflectance, sun glints, spectral index

## Abstract

This paper analyzes the digital modelling of radiance reflectance of the sea surface when the water column is polluted by oil-in-water emulsion. A method tracking the fate of two billion virtual solar photons was applied to obtain the angular distribution of bottom-up radiance for a plane of sunlight striking the sea surface. For the calculations, the inherent optical properties of seawater characteristic for the Gulf of Gdańsk (southern Baltic Sea) were used. The analyses were performed for two types of oils with extremely different optical properties for an oil concentration of 10 ppm and for a roughened sea surface with a wind speed of 2 m/s. The spectral index for oil detection in seawater for different viewing angles was determined based on the results obtained for reflectance at eight wavelengths in the range of 412–676 nm for viewing angle in the range from 80° to 0°, both on the side of incidence of direct sunlight and on the opposite side. The resulting calculated spectral indexes for different wavelength combinations indicated significant dependence on the viewing angle.

## 1. Introduction

Oil substances in the marine environment may appear on the sea surface in the form of varying oil layer thicknesses in a water-in-oil emulsion or a monomolecular oil film [1]. There are numerous methods for remotely detecting such contaminants, both active and passive. If passive methods are considered, surface oil sensing devices used for decades in aerial surveillance include ultraviolet (UV) and infrared (IR) scanners. In those techniques, images in UV deliver information about the extent of the oil spot, while IR images provide information about its thickness. In techniques of oil detection using the visible range (VIS), the observer’s eye, both human and a photo-camera, must be considered [2]. For several years, radar installed on aircrafts and satellites—synthetic aperture radar (SAR)—has been used with quite good results [3]. For example, in Europe, the European Maritime Safety Agency (EMSA) [4] uses this method. For the Baltic Sea, the Helsinki Commission (HELCOM) gathers information about oil spills in this area [5].

Scientific literature describing various methods of the sea surface oil detection is extensive, and its synthesis and indications of the scope of operational applications have been presented in publications and a monograph by Fingas [6,7,8]. However, the surface forms of the oil can evolve into underwater forms. This happens as a result of the action of environmental forces and the consequence of physicochemical changes of oil as a result of the passage of volatile oil components into the atmosphere and the transfer of water-soluble components to the water column [1,9]. An intentional action, such as the use of chemical agents to disperse oil in the water, cannot be excluded [10,11,12]. When pollution is spread in the water column, it can only be seen in the visible range because other types of electromagnetic radiation are absorbed in water. Depending on the region of the sea, a certain amount of light may penetrate to a depth of several to several dozen meters, and then partially return to the atmosphere, spectrally changed as a result of interactions with water and its components, not excluding possible oil pollution. 

In remote maritime research, researchers are generally interested in tracking the biological component [13], as well as the methodology, of such research, including the participation of various suspensions in shaping the light field in and above the water. Oil dispersed in water can form a relatively stable system known as oil-in-water emulsion. Disregarding the presence of oil substances in the sea can negatively affect the effectiveness of algorithms used in marine bio-optics, due to how the oil affects the spectral composition of the light leaving the sea surface.

The modification of the light field above the sea surface has been demonstrated in studies of optical contrast between the surface of clean and polluted seawater with dispersed oil [14,15]. The scope of this paper is a description of the preparation of the theoretical basis for designing an optical sensor that can be used to remotely detect oil residing below the sea surface. The fact that oil affects the spectral composition of light leaving seawater has been demonstrated in studies of the impact of light wavelength on remote sensing reflectance (*R_rs_*) for seawater polluted by an oil-in-water emulsion [16]. It was noted that the specific combinations of two wavelengths could be used to create an index whose value would allow focusing on the presence of oil below the water surface. It should be noted that the values of spectral indexes were determined for the radiance reflectance (*R_L_*) measured perpendicular to the sea surface in operational oceanography, referred to as remote sensing reflectance (*R_rs_*). However, there may be occasions when the radiation sensor can be set to various nadir angles. The question arises as to what extent the viewing angle can affect the effectiveness of detecting oil pollution in water. This study is a continuation and widening of earlier performed spectral analysis of remote sensing reflectance (*R_rs_*) of the sea area polluted with dispersed oil [16]. In this paper, we report the results of spectral index analyses determined over a wide range of viewing nadir angles in the plane of direct sunlight, as opposed to the previous work [16] in which analyses were carried out for only one viewing angle (0°).

## 2. Materials and Methods

The study used the method of simulating the trajectories of a large number of virtual photons belonging to the Monte Carlo group of methods used to model the light field (angular distribution of radiance) in the water column and above the sea surface. The code, used for the Monte Carlo numerical runs, was written in Ansi C by Piskozub (co-author) and has been used in multiple studies of the light field, both in the marine environment and inside optical instruments [17,18,19,20,21]. The code involves forward tracing of virtual “photons” in layered marine environment. The code has an air–sea interface obeying Snell and Fresnel laws, as well as atmospheric layers. All layers have prescribed absorption and scattering values with the possibility of including multiple types of scatterers with different scattering phase functions. Photons are traced in a stochastic way using pseudo random numbers, probability distributions of the pathways (based on the absorption and scattering values), and scattering angles in 3D (based on the phase functions used). The code allows for any combinations of angular sector receivers at any depth, making it possible to record not only irradiance and radiance values, but even bidirectional reflectance distribution functions (BRDF). Optical models of unpolluted and polluted sea basin, as well as the model of hemisphere (direct solar light and light from the entire sky), were used in the same way as in the authors’ previous study on sensing oil dispersed below the sea surface [16]. In particular, an optical model of the sea with a relatively high content of suspensions was used to investigate the extent to which the light coming out of the sea reflected the influence of alien substances. The data of the optical properties of oil-free seawater were taken from the results of several years of spectral measurements of the absorption coefficient distributions and the scattering coefficient in the Gulf of Gdańsk (southern Baltic Sea) [22]. The research included optical parameters of two types of oils with extremely different refractive indexes and light absorption coefficient (Petrobaltic with relatively good transparency, relatively low refractive index, and opaque Romashkino with a higher refractive index). The virtual sun was placed at an angle of 30 degrees. The modelled sea was 100 m deep with a bottom, with an albedo of 0.10 (0.02 specular and 0.08 diffusive). It was divided into three layers: 0–5 m, 5–30 m, and 30–100 m. The two top layers were assumed to be polluted with oil. The angular distribution of scattering on water was represented by coastal Petzold phase function [23], and scattering on oil was represented with oil phase functions. The values of total absorption and scattering for each of the two scattered types are given in Tables 2–4 of [16]. The phase function for the oil-in-water emulsion was calculated by Otremba and Piskozub using the Mie solution [24] for the experimentally determined size distribution of oil droplets in an artificially produced emulsion stored for 7 days. The phase functions for fresh and older emulsions differed little over a wide range of scattering angles. Each run consisted of 2 billion (2 · 10^9^) virtual photons. The run time depended on the actual absorption and scattering values, but generally it took less than 72 h on a modern desktop PC. The single run time depended on the actual absorption and scattering values; however, generally, it took less than 72 h on a desktop PC (16-core processor Advanced Micro Devices (AMD) Ryzen 9, RAM 32 GB, 64-bit operating system Windows 10 Pro). Each solar photon falling on the surface of the sea has a chance to penetrate deep into the water to return to the atmosphere after a certain journey. Some photons do not pass into the water since they are reflected from the surface. Most water-penetrating photons are absorbed in the water column—which ends their history. The number of photons returning to the atmosphere depends on the impact of the sea components. Both suspended and dissolved components of the seawater, as well as density fluctuation of water, influence the direction in which they are possibly directed towards the atmosphere. In any case, to obtain the spatial distribution of photons above the sea surface, it is necessary to trace the fate of a very large number of solar photons. In our earlier study [16], only photons perpendicularly passing into the atmosphere were counted, and the fates of 200 million virtual solar photons falling on the sea surface were examined. A virtual receiver of water-leaving photons registered them from a solid angle of 0.0157 sr, which corresponded to about an 8-degree cone opening. This corresponded to a situation in which the sensor registered radiance from such a solid angle [25]. The radiance for a defined wavelength is a mathematical quantity in which the infinitesimal size of the solid angle *dΩ* occurs (Formula (1)).

(1) $$L(\theta ,\varphi )=\frac{dF(\theta ,\varphi )}{dA\cos\theta d\Omega (\theta ,\varphi )}$$

where *dF* is infinitesimal flux of light energy emitted from infinitesimal sea surface *dS* into infinitesimal solid angle dΩ along direction *θ* (zenith), *φ* (azimuth). Since the value of radiance depends on the intensity of sunlight in the marine optics, the concept of radiance reflectance *R_L_* is used, as *L* divided by solar downward vector irradiance *E* (W m^−2^). Therefore, the reflectance unit is sr^−1^. In the computer simulation of virtual photon migration, the role of *dF/(dA* cos*θ)* is played by the number of photons *N* counted in a specific sector of the small solid angle Ω. However, the role of irradiance is played by the number of virtual photons *N_o_* falling on the sea surface from the upper hemisphere.

In the radiance meter, *dF* is represented by the energy of light reaching the photosensitive element from a defined small solid angle, while *dA* is represented by the size of the sea surface from which energy is collected. Therefore, in the practice of in situ measurement and in a computer simulation, this solid angle must have the smallest value. However, minimizing the viewing angle in the radiation meter leads to a decrease in measurement accuracy because the light sensor in the radiance meter reaches a small number of photons. The same applies to the virtual photon receiver in simulation measurements. In principle, the viewing angle in simulations can be arbitrarily small, although it forces the use of a large number of testing of virtual photons, and this extends the measurement time (up to several days using a personal computer).

**Figure 1:**
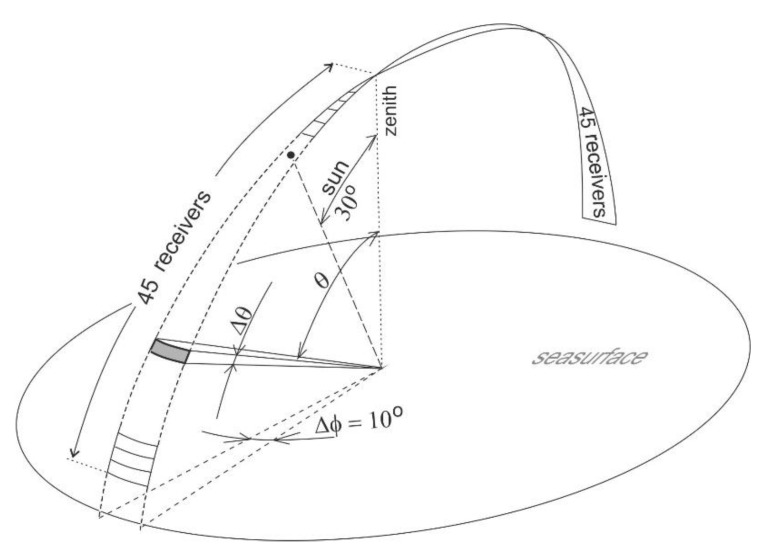
Geometry of virtual measurement of upward radiance.

In this study, photons moving in the atmosphere in different directions were counted in receiver sectors with very small solid angle values—from 0.000436 sr to 0.00436 sr. Ninety receivers were used in an angle range from −80.6 to +80.6 degrees in the plane of incidence of sunlight. The zenith angles measured from the incident light directly from the sun were defined as negative, and from the opposite side as positive. The geometry of the virtual measurement of the distribution of upward radiance determined above the sea surface is shown in Figure 1. The angular boundaries of individual sectors, and their sizes expressed as solid angle values, are listed in Table 1. In order to minimize the spread of photons recorded, the number of solar photons *N_o_* was increased to 2 billion.

## 3. Results and Discussion

**Figure 2:**
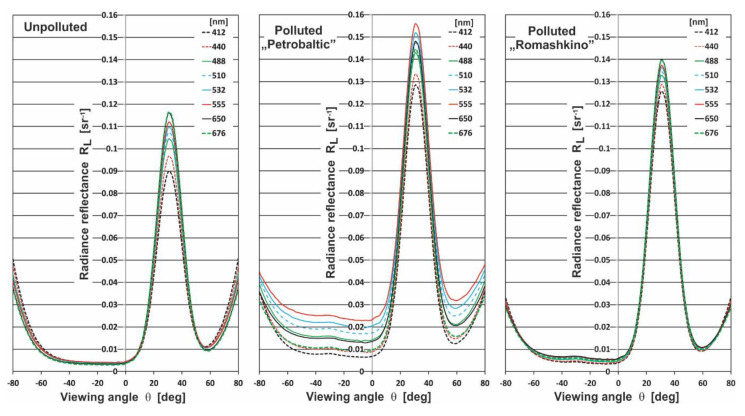
The radiance reflectance R_L_ above sea surface as a function of viewing angle *θ* for selected wavelengths, 412 nm, 440 nm, 488 nm, 510 nm, 532 nm, 555 nm, 650 nm, and 576 nm, respectively, and *θ* changes in the range from −80° to 80° for natural seawater (unpolluted) and seawater polluted by Petrobaltic and Romashkino oil for 10 ppm oil concentration.

Figure 2 presents the radiance reflectance (*R_L_*) for selected wavelengths at 412 nm, 440 nm, 488 nm, 510 nm, 532 nm, 555 nm, 650 nm, and 676 nm, respectively, as a function of viewing angle *θ* for natural (unpolluted) seawater and the same seawater polluted by Petrobaltic and Romashkino oil. The calculations were performed for an oil concentration of 10 ppm in the case of waved sea surface with a wind speed of 2 m/s (according to the Cox and Munk algorithm [26]). The differences in *R_L_* for unpolluted seawater and seawater polluted by oil in Figure 2 are clear. Moreover, a dependence in the values of *R_L_* was detected for two optically different kinds of oils. The *R_L_* achieved higher values for Petrobaltic crude oil, which were caused due to lower values of absorption coefficients a_o_(λ) and higher values of scattering coefficients b_o_(λ) for Petrobaltic than for Romashkino crude oil [15]. Figure 2 shows the specific choice of *R_L_* values for viewing angle *θ* in the range from 10° to 50°, with the *R_L_* maximum at 30° (reflection direction of solar rays if the sea surface is flat).

Typical remote sensors measure remote-sensing reflectance *R_rs_* as the ratio of the above-water upwelling radiation *L_u_* in the viewing angle 0° to the above-water down-welling vector irradiance E_d_. Moreover, the satellite sensor algorithms are based on the ratios of R_rs_ (λ) for selected wavelengths mainly to determine the chlorophyll concentration [27]. Therefore, the intention of this study was to find an efficient spectral index based on a wavelength combination of radiance reflectance *R_L_* for oil detection considering a different viewing angle *θ*. The spectral index *I* (*θ*) was defined as the ratio of reflectance for the longer wavelengths, to the reflectance for the lower wavelengths for the selected viewing angle *θ* (Formula (2)):

(2) $$I(\theta )=\frac{R_{L}\left(\lambda_{n},\theta \right)}{R_{L}\left(\lambda_{m},\theta \right)}$$

where *λ_n_*, *λ_m_* = higher wavelength and lower wavelength.

**Figure 3:**
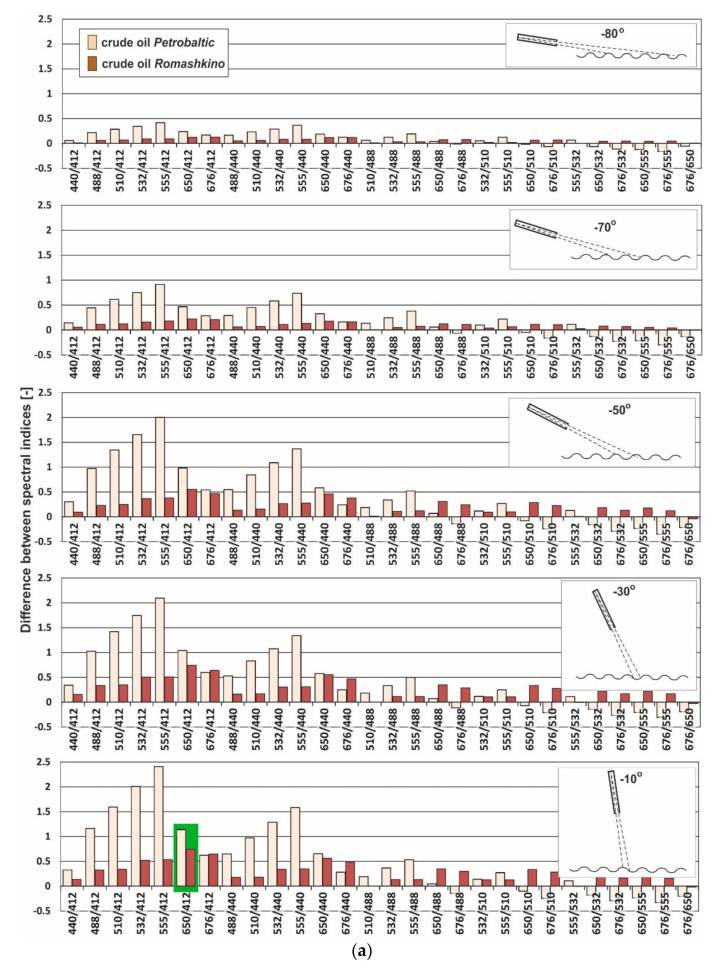

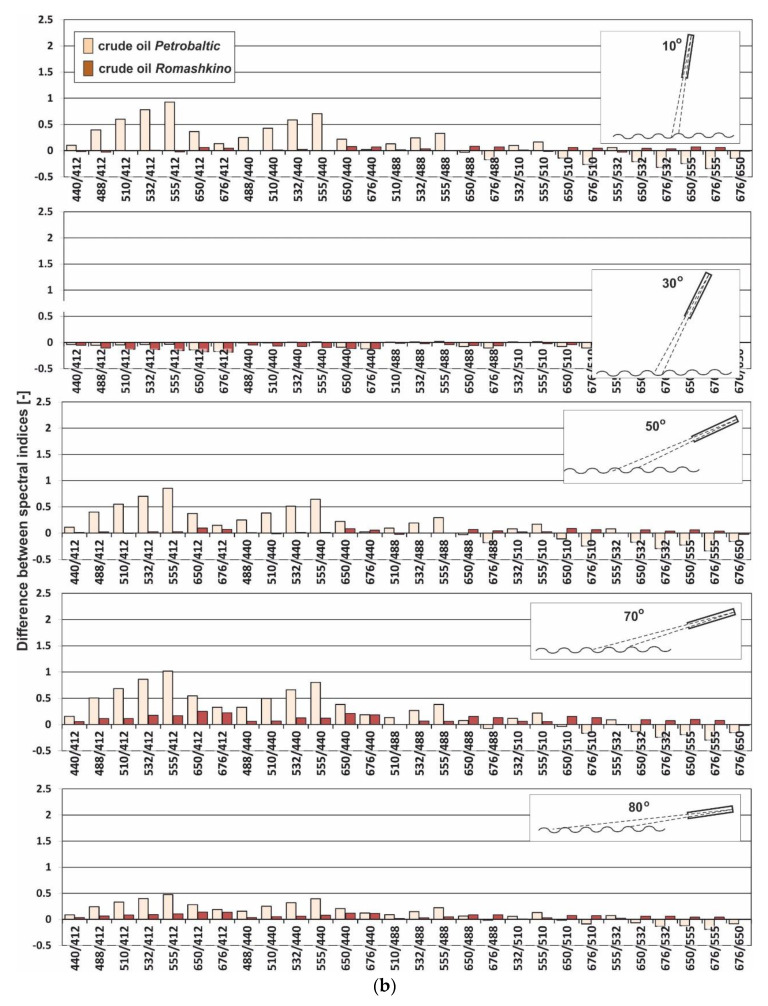
(**a**) Differences between spectral indices of water contaminated by dispersed oil, and indices of oil-free water, determined at chosen viewing angles measured from the solar light side (−80°, −70°, −50°, −30°, and −10°, respectively), for roughened sea surface with a 2 m/s wind speed. The green rectangle on the bottom chart indicates an optimal for the analyzed case viewing angle and wavelength combination for the spectral index. (**b**) Differences between spectral indices of water contaminated by dispersed oil and indices of oil-free water, determined at chosen viewing angles measured on the opposite of solar light side (10°, 30°, 50°, 70°, and 80°, respectively), for roughened sea surface with a 2 m/s wind speed.

The spectral index was determined as a combination of *R_L_* for 412 nm, 440 nm, 488 nm, 510 nm, 532 nm, 555 nm, 650 nm, and 676 nm wavelengths, for natural seawater and seawater polluted by crude oil. The calculations of the spectral index were performed for the selected viewing angle *θ*. Figure 3a presents values of the spectral index for all combinations of wavelengths for Petrobaltic and Romashkino crude oils, for viewing angles of −10°, −30°, 50°, −70°, and −80°, respectively, and Figure 3b presents values of the spectral index for all combinations of wavelengths for Petrobaltic and Romashkino crude oils, for viewing angles of 10°, 30°, 50°, 70°, and 80°, respectively. 

**Figure 4:**
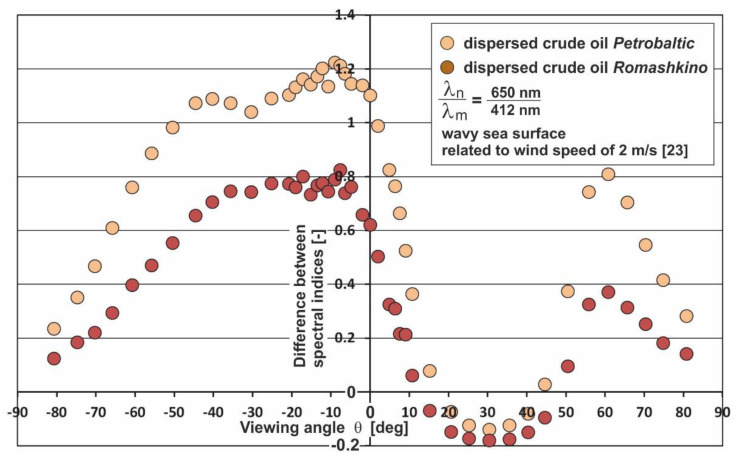
Viewing angle dependence of differences between spectral indices of seawater contaminated by dispersed oil and indices of seawater free of oil contamination, for chosen wavelengths λ_n_ and λ_m_ (650 nm and 412 nm).

Given both the high index values and the small differences between the index values for both types of oil, the index for 650/412 wavelengths seemed to be optimal. The index for that wavelengths combination was selected while taking into account the comparison of the index values for pure (natural) seawater with those of the oil polluted seawater. In relation to the pure seawater for those wavelength combinations, the index for contaminated seawater achieved higher values than for pure (natural) seawater (the comparison is presented in Figure 3). Moreover, that selection of the index is a result of its being sensitive to oils with optical properties of the Petrobaltic type (with a low value of the absorption coefficient and refractive index), as well as to oils with optical properties of the Romashkino type oil (with a high value of the absorption coefficient and refractive index). Furthermore, due to the completely opposite optical properties of oils, the spectral selected index allows any oil to be detected. The dependence of the spectral index for two kinds of oils on the viewing angle *θ* is presented in Figure 4, which shows that oil detection is optimal in the *θ* range from −50° to 5°, and from 50° to 70°. 

In the case when, in a given region of the sea, oil contamination could occur with optical properties similar to Petrobaltic crude oil, 555/412 would be an adequate indicator. The selection of this index is due to the difference of its value in relation to the pure seawater, which is always the greatest regardless of the viewing angle. At the same time, it is worth noting that within the viewing angles from 10° to 50° with such an index, it will not be possible to detect oil with optical properties similar to Romashkino crude oil. Otherwise, a better solution would be to use a proper indicator for detecting an oil optically similar to Romashkino crude oil, provided that other oils are also noted. Therefore, this case corresponded to the index 650/412. Since Petrobaltic and Romashkino are oils with diametrically different optical properties, it should be expected that other oils will also be detected with this optical index.

The method described in the paper was less sensitive than laboratory chemical methods [28], although it required seawater sampling. It was also possible to construct an oil immersion sensor based on the phenomenon of fluorescence [29]. However, the advantage of the analyzed method was the possibility to detect an oil emulsion in the seawater column by means of a sensor that analyzed the light coming from the sea surface, set at the right angle.

## 4. Conclusions and Outlook

Radiance reflectance (*R_L_*) modelling for various viewing angles in the range from 80° to 0°, both on width of incidence of direct sunlight and on the opposite side, was performed to determine the optimal spectral index used for a remote sensor to detect oil polluting the seawater column. The calculations were performed for the optical properties of an example sea basin (the Gulf of Gdańsk), assuming natural seawater and the same seawater polluted with dispersed oil for an oil concentration of 10 ppm, and for a roughened sea surface with a wind speed of 2 m/s. The obtained data indicated the strong dependence of *R_L_* on the viewing angle. Significant changes in the values of *R_L_* with the viewing angle were observed for Petrobaltic crude oil—a substance characterized by relatively low light absorption and a low refractive index—opposite to Romashkino crude oil. Moreover, the calculated spectral indexes indicated a dependence on the viewing angle (*θ*), with differences if applied to two kinds of oils. These results indicated that oil detection would be optimal in the *θ* range from −50° to 5° and from 50° to 70° (while the angle of incidence of direct solar rays is −30°), for any possible type of oil dispersed in seawater. Considering the detection of oil at any viewing angle, the spectral index for the combination of wavelengths 650/412 was most useful, noting at the same time that all combinations of wavelengths related to short wavelengths (in the analyzed case of 412 and 440 nm) were “sensitive” to oil with any optical parameters, which was present in the sea column as oil-in-water emulsion. In the next work, we are planning to model radiance reflectance in the search for the optimal viewing angle within the entire hemisphere. In the future, we also plan to focus on the detection of dispersed oil under a cloudy sky and to assess other types of seawater.

## Figures and Tables

**Table 1:** The angular boundaries of individual sectors and the sector dimensions expressed as solid angle values.

Sector Dimension [sr]	Sector Borders [deg]	Sector Dimension [sr]
0.000436	0	
	4.05	
	5.73	
	7.02	
	8.11	0.000873
	9.94	
	11.48	
	12.84	
0.001745	14.07	
	16.26	
	18.20	
	19.95	
	21.57	
	23.07	
	24.49	
	25.84	0.004363
	28.96	
	31.79	
	34.41	
	36.87	
	39.20	
	41.41	
	43.53	
	45.57	
	47.54	
	49.46	
	51.32	
	53.13	
	54.90	
	56.63	
	58.33	
	60.00	
	61.64	
	63.25	
	64.85	
	66.42	
	67.98	
	69.51	
	71.04	
	72.54	
	74.04	
	75.52	
	77.00	
	78.46	
	79.92	
	81.37	
